# Laboratory identification and clinical characteristics of *Streptococcus bovis*/*Streptococcus equinus* complex bacteremia: a retrospective, multicenter study in Hiroshima, Japan

**DOI:** 10.1186/s12879-021-06880-4

**Published:** 2021-11-26

**Authors:** Yuki Kaiki, Hiroki Kitagawa, Kayoko Tadera, Hiroyuki Taogoshi, Mitsuyasu Ikeda, Mikihiro Kano, Toshie Harino, Toshihito Nomura, Keitaro Omori, Norifumi Shigemoto, Shinya Takahashi, Hiroki Ohge

**Affiliations:** 1 Department of Surgery, Graduate School of Biomedical and Health Sciences, Hiroshima University, Hiroshima, Japan; 2 Department of Infectious Diseases, Hiroshima University Hospital, Hiroshima, Japan; 3 Project Research Center for Nosocomial Infectious Diseases, Hiroshima University, Hiroshima, Japan; 4 Section of Clinical Laboratory, Division of Clinical Support, Hiroshima University Hospital, Hiroshima, Japan; 5 Division of Laboratory Medicine, Hiroshima University Hospital, Hiroshima, Japan; 6 Department of Surgery, JA Hiroshima General Hospital, Hiroshima, Japan; 7 Present Address: Section of Clinical Research and Laboratory, JA Hiroshima General Hospital, Hiroshima, Japan; 8 Department of Surgery, Hiroshima City Asa Citizens Hospital, Hiroshima, Japan; 9 Department of Clinical Laboratory, Hiroshima City Asa Citizens Hospital, Hiroshima, Japan; 10 Translational Research Center, Hiroshima University, Hiroshima, Japan

**Keywords:** *Streptococcus bovis*, Bloodstream infection, MALDI-TOF MS, *sodA*, Colorectal cancer, Infective endocarditis

## Abstract

**Background:**

Bacteremia due to the *Streptococcus bovis*/*Streptococcus equinus* complex (SBSEC) is associated with specific diseases, such as colorectal cancer and infective endocarditis. This study aimed to evaluate the clinical characteristics of SBSEC bacteremia and the accuracy of identification of matrix-assisted laser desorption/ionization time-of-flight mass spectrometry (MALDI-TOF MS) and phenotypic identification systems for SBSEC isolates.

**Methods:**

We analyzed patients with SBSEC bacteremia retrospectively between 2012 and 2019 at three hospitals in Japan. We re-identified each SBSEC isolate using sequencing superoxide dismutase (*sodA*) analysis, MALDI-TOF MS using the MALDI Biotyper, and phenotypic identification using the VITEK2.

**Results:**

During the study period, 39 patients with SBSEC bacteremia were identified. *S. gallolyticus* subsp. *pasteurianus* (SGSP, *n* = 29), *S. gallolyticus* subsp. *gallolyticus* (SGSG, *n* = 5), *S. lutetiensis* (SL, *n* = 4), and *S. infantarius* subsp. *infantarius* (*n* = 1) were identified using *sodA* sequencing analysis. Primary bacteremia (36%) was the most common cause of bacteremia, followed by infective endocarditis (26%) and biliary tract infections (23%). Colorectal cancer was associated significantly with SGSG bacteremia, while the sources of bacteremia were similar in each SBSEC subspecies. The MALDI Biotyper was significantly more accurate in identifying the SBSEC isolates at the subspecies level compared to the VITEK2 (92% vs. 67%, *P* = 0.010). In contrast, there were no significant differences in the rates of correct identification of the SBSEC isolates at the species level between the MALDI Biotyper and the VITEK2 (100% vs. 87%, *P* = 0.055).

**Conclusions:**

Bacteremia with SGSG was associated with colorectal cancer, and the sources of bacteremia were similar in each SBSEC subspecies. The MALDI-TOF MS was significantly more accurate in identifying SBSEC isolates at the subspecies level than the phenotypic identification systems. The accurate identification of SBSEC isolates using the MALDI-TOF MS and phenotypic identification systems was sufficient at the species level, but it was insufficient at the subspecies level. Therefore, it may be reasonable for clinicians to perform echocardiographies and colonoscopies in all patients with SBSEC bacteremia.

## Background

*Streptococcus bovis*/*Streptococcus equinus* complex (SBSEC) is common inhabitants of the digestive tract in animals and humans. The introduction of new nomenclature of SBSEC and development of molecular techniques for identification have revealed specific disease associations with different SBSEC species and subspecies [1–3]. *Streptococcus gallolyticus* subsp. *gallolyticus* (SGSG) bacteremia is associated with infective endocarditis and colorectal cancer [4, 5]. *Streptococcus gallolyticus* subsp. *pasteurianus* (SGSP) and *Streptococcus infantarius* are associated with biliary tract infections and biliary-pancreatic cancer [6–9]. Therefore, an accurate identification of SBSEC at the subspecies level may be required. The most reliable methods currently available for identifying SBSEC species are gene-based methods, such as partial sequences of *sodA*, *gyrB*, and *groE* [10]. However, phenotypic identification systems remain standard in clinical laboratories. Matrix-assisted laser desorption/ionization-time of flight mass spectrometry (MALDI-TOF MS) has been used widely as an alternative for bacterial identification in clinical microbiology and infectious disease fields. However, only a few studies have evaluated the performance of MALDI-TOF MS in the accurate identification of the species and subspecies of SBSEC [11–13]. Additionally, SBSEC infections are limited and have not been investigated fully [14, 15]. Therefore, this study aimed to identify the clinical characteristics of SBSEC bacteremia in Japanese patients and evaluate the accuracy of identification of the MALDI-TOF MS system (MALDI Biotyper) and phenotypic identification system (VITEK2), using *sodA* sequencing as the reference standard.

## Methods

### Study design and obtained data

A retrospective, multicenter, case-control study was conducted at the Hiroshima University Hospital, Hiroshima General Hospital, and Hiroshima City Asa Citizens Hospital in Hiroshima, Japan. This study recruited all the patients with SBSEC bacteremia admitted between January 1, 2012, and December 31, 2019, at these hospitals. The patients were identified using the microbiology database of each institution. SBSEC bacteremia was defined based on the isolation of the organism from one or more sets of blood culture bottles. The study protocol was approved by the Institutional Review Boards of each institution. The requirement for written informed consent was waived due to the retrospective study design. The patient data obtained from the medical records included age, sex, comorbidities, surgical histories, sources of bacteremia, whether colonoscopies or echocardiographies were performed during the treatment period, and the 30-day all-cause mortality. If the source of the infection was not identified, the bacteremia was classified as a primary bacteremia.

### Identification of SBSEC

The SBSEC isolates were obtained by processing the blood culture samples in the BacT/ALERT 3D (bioMérieux, Marcy l’Étoile, France) at the Hiroshima University Hospital and Hiroshima City Asa Citizens Hospital. In the Hiroshima General Hospital, the blood culture system that was used between 2012 and 2014 was the BacT/ALERT 3D, and between 2015 and 2019, the VersaTREK (Thermo Scientific, Westlake, OH). SBSEC was identified using an automated phenotypic identification system VITEK2 Compact (bioMérieux) with a VITEK-2 GP ID card (bioMérieux), according to the recommendations of the manufacturer, at the Hiroshima University Hospital and Hiroshima General Hospital. In the Hiroshima City Asa Citizens Hospital, SBSEC was identified using the Rapid ID32 Strep (bioMérieux). The SBSEC isolates were stored as glycerol stocks at − 70 °C until use. Species re-identification of SBSEC isolates was performed using sequencing *sodA*, MALDI-TOF MS using the MALDI Biotyper, and phenotypic identification using the VITEK2 Compact at the Hiroshima University Hospital. For the *sodA* sequencing analysis, bacterial DNA was extracted using the QIAamp DNA Blood and Tissue Mini Kit (Qiagen, Germany). The *sodA* PCR was performed as described previously [16]. The amplicons were sequenced using the ABI Prism 3130xl Genetic Analyzer (Applied Biosystems, Foster City, CA) with a Big-Dye kit (Applied Biosystems). The amplified sequences were compared with reference sequences from the GenBank database using a BLAST search (https://www.ncbi.nlm.nih.gov/BLAST/). A sequence similarity of 99% was used as a “cutoff” value for the SBSEC species identification [17].

A MALDI-TOF MS analysis was performed using a BD MALDI Biotyper Sirius system (Becton, Dickinson and Company, USA) with the MBT Compass 4.1 that included the MBT Compass library Ver.9.0.0.0 (8468MSPs) (Bruker Daltonik GmbH, Bremen, Germany). Bacteria were cultured on 5% sheep blood agar (Eiken Chemical, Tokyo, Japan) in 5% CO_2_ at 37 °C for 24 h. A MALDI-TOF MS analysis using pure culture colonies was performed using direct transfer methods, as described previously [18]. As specified by the manufacturer, identification scores ≥ 2.0 were accepted for reliable identification at the species/subspecies level. The results with the highest scores were reported.

### Statistical analysis

The continuous variables were analyzed using the Wilcoxon rank-sum test. The categorical variables were analyzed using the χ^2^ test or Fisher’s exact test for small samples. All the statistical analyses were performed using JMP 16.0 (SAS Institute Inc., Cary, NC, USA). All the *P* values were two-sided, and statistical significance was set at *P* < 0.05.

## Results

### Clinical characteristics of patients with SBSEC

During the study period, 39 patients with SBSEC bacteremia were identified (Hiroshima University Hospital, 25 cases; Hiroshima General Hospital, nine cases; and Hiroshima City Asa Citizens Hospital, five cases). Of these, SGSP (*n* = 29, 75%), SGSG (*n* = 5, 13%), *S. lutetiensis* (SL, formerly *S. infantarius* subsp. *coli*) (*n* = 4, 10%), and *S. infantarius* subsp. *infantarius* (SISI) (*n* = 1, 3%) were identified using the *sodA* sequencing analysis.

The demographic and clinical characteristics of the patients with SBSEC bacteremia are summarized in Table 1. The median age of the patients was 76 (range 28–92) years. There were 25 men (64%). One pregnant woman with SGSP bacteremia underwent cesarean section, followed by heart valve replacement surgery. There were no pediatric patients (aged < 18 years). Primary bacteremia was the most common source of SBSEC bacteremia (*n* = 14, 36%), followed by infective endocarditis (*n* = 10, 26%) and biliary tract infections (*n* = 9, 23%). There were no significant differences in the frequencies of the source of bacteremia among the SBSEC subspecies isolates (only the statistical data of SGSP and SGSG are shown in Table 1). Of all patients, 54%, 41%, and 95% underwent transthoracic electrocardiographies, colonoscopies, and computed tomography (CT) scans, respectively. Overall malignancies were diagnosed in 54% of patients with SBSEC bacteremia. There were no significant differences in the incidence of overall malignancies among each SBSEC subspecies (data not shown). However, the incidence of overall colorectal cancer and colorectal cancer identified after the diagnosis of bacteremia in patients with SGSG bacteremia was significantly higher than that in patients with SGSP bacteremia (*P* = 0.048 and 0.015, respectively). In addition, the incidence of overall colorectal cancer in patients with SGSG bacteremia was significantly higher than that in patients with bacteremia due to other SBSEC subspecies (*P* = 0.049). The overall 30-day mortality rate was 13% (5 of 39 cases); all five patients had SGSP bacteremia and two of them had polymicrobial bacteremia.

**Table 1:** Demographic and clinical characteristics of patients with bacteremia due to *Streptococcus bovis*/*Streptococcus equinus* complex

Characteristics	*S. gallolyticus* subsp. *pasteurianus* (*n* = 29)	*S. gallolyticus* subsp. *gallolyticus* (*n* = 5)	*S. lutetiensis* (*n* = 4)	*S. infantarius* subsp. *infantarius* (*n* = 1)	Total (*n* = 39)	*P* value SGSP vs. SGSG
Age, median (range)	74 (28–92)	81 (69–83)	82 (77–92)	91	76 (28–92)	0.39
Gender, men (%)	18 (62)	4 (80)	3 (75)	0 (0)	25 (64)	0.63
Pregnant women	1 (3)	0 (0)	0 (0)	0 (0)	1 (3)	1
Co-morbidities						
Diabetes mellitus	7 (24)	0 (0)	1 (25)	0 (0)	8 (21)	0.56
Liver cirrhosis	2 (7)	0 (0)	0 (0)	0 (0)	2 (5)	1
Chronic kidney disease	1 (3)	1 (20)	1 (25)	1 (100)	4 (10)	0.28
Neutropenia at culture	3 (10)	0 (0)	0 (0)	0 (0)	3 (8)	1
Gastrointestinal surgery within 30 days	0 (0)	0 (0)	0 (0)	0 (0)	0 (0)	1
Malignancies						
Overall malignancies	16 (55)	4 (80)	1 (25)	0 (0)	21 (54)	0.38
Gastrointestinal tract						
Esophageal	3 (10)	0 (0)	0 (0)	0 (0)	3 (8)	1
Stomach	1 (3)	1 (20)	0 (0)	0 (0)	2 (5)	0.28
Colon/rectum	4 (14)	3 (60)	1 (25)	0 (0)	8 (21)	0.048
Bile duct	3 (10)	0 (0)	0 (0)	0 (0)	3 (8)	1
Liver	1 (3)	0 (0)	0 (0)	0 (0)	1 (3)	1
Other malignancies						
Hematologic	4 (14)	1 (20)	0 (0)	0 (0)	5 (13)	1
Brain	1 (3)	0 (0)	0 (0)	0 (0)	1 (3)	1
Oral cavity	3 (10)	0 (0)	0 (0)	0 (0)	3 (8)	1
Prostate	2 (7)	0 (0)	0 (0)	0 (0)	2 (5)	1
Ovary	2 (7)	0 (0)	0 (0)	0 (0)	2 (5)	1
Multiple malignancies (> 1)	7 (24)	1 (20)	0 (0)	0 (0)	8 (21)	1
Known before the bacteremia	13 (45)	1 (20)	1 (25)	0 (0)	15 (38)	0.38
Discovered after the bacteremia	3 (10)	3 (60)	0 (0)	0 (0)	6 (15)	0.029
Colon/rectum	2 (7)	3 (60)	0 (0)	0 (0)	5 (13)	0.015
Bile duct	1 (3)	0 (0)	0 (0)	0 (0)	1 (3)	1
Colorectal cancer						
Known before the bacteremia	2 (7)	0 (0)	1 (25)	0 (0)	3 (8)	1
Discovered after the bacteremia	2 (7)	3 (60)	0 (0)	0 (0)	5 (13)	0.015
Endoscopic examination performed following the bacteremia	10 (34)	3 (60)	2 (50)	1 (100)	16 (41)	0.35
Source of bacteremia						
Primary bacteremia	10 (34)	1 (20)	2 (50)	1 (100)	14 (36)	1
Infective endocarditis	7 (24)	2 (40)	1 (25)	0 (0)	10 (26)	0.59
Biliary tract infection	6 (21)	2 (40)	1 (25)	0 (0)	9 (23)	0.57
Urinary tract infection	2 (7)	0 (0)	0 (0)	0 (0)	2 (5)	1
Others	4 (14)	0 (0)	0 (0)	0 (0)	4 (10)	1
Infective endocarditis						
TTE performed following the bacteremia	16 (55)	2 (40)	3 (75)	0 (0)	21 (54)	0.65
TEE performed following the bacteremia	0 (0)	2 (40)	1 (25)	0 (0)	3 (8)	0.018
Computed tomography	27 (93)	5 (100)	4 (100)	1 (100)	37 (95)	1
Polymicrobial bacteremia	6 (21)	0 (0)	1 (25)	0 (0)	7 (18)	0.56
*Escherichia coli*	3 (10)	0 (0)	0 (0)	0 (0)	3 (8)	1
*Klebsiella pneumoniae*	1 (3)	0 (0)	0 (0)	0 (0)	1 (3)	1
*Corynebacterium jeikeium*	1 (3)	0 (0)	1 (25)	0 (0)	2 (5)	1
*Streptococcus pneumoniae*	1 (3)	0 (0)	0 (0)	0 (0)	1 (3)	1
Thirty-day all-cause mortality	5 (17)	0 (0)	0 (0)	0 (0)	5 (13)	1

*TEE* transesophageal echocardiography; *TTE* transthoracic echocardiography; *SGSP Streptococcus gallolyticus* subspecies *pasteurianus*; *SGSG Streptococcus gallolyticus* subspecies *gallolyticus*

### Identification of SBSEC

The identification results of 39 isolates from patients with SBSEC bacteremia are shown in Table 2. The MALDI Biotyper correctly identified all the SBSEC isolates at the species level and 92% (36 of 39) at the subspecies level, which was confirmed using *sodA* sequencing. In the identification process using MALDI Biotyper, the identification results of all SBSEC isolates ranked 1–10 were SBSEC species. In 19 of 39 isolates, the results of the identification with score ≥ 2.0 using the MALDI Biotyper included other SBSEC species. The MALDI Biotyper identified all SGSPs at the subspecies level correctly. However, only 80% of the SGSG and 75% of the SL isolates were identified correctly at the subspecies level. One isolate each of SGSG and SL were misidentified as SGSP, and one SISI isolate was misidentified as *S. equinus*.

**Table 2:** Comparison of identification results of 39 isolates of SBSEC using the MALDI Biotyper and VITEK2 compact

SBSEC type	Reference (*sodA*), n	MALDI Biotyper			VITEK2				
		Identified with highest score as^a^	Correct identification to the subspecies level	Correct identification to the SBSEC species level	Identified with high probability as^b^	Low discrimination	Not identified	Correct identification to the subspecies level	Correct identification to the SBSEC species level
SGSP	29	29 SGSP	29 (100)	29 (100)	20 SGSP	5 (17) ^c^	4 (14)	20 (69)	24 (83)
SGSG	5	4 SGSG, 1 SGSP	4 (80)	5 (100)	5 SGSG	0 (0)	0 (0)	5 (100)	5 (100)
SL	4	3 SL, 1 SGSP	3 (75)	4 (100)	1 SL, 1 SGSP	2 (50)^d^	0 (0)	1 (25)	4 (100)
SISI	1	1 SE	0 (0)	1 (100)	None	1 (100)^e^	0 (0)	0 (0)	1 (100)
Total	39	–	36 (92)*	39 (100)^†^	–	8 (21)	4 (10)	26 (67)*	34 (87)^†^

*SBSEC Streptococcus bovis*/*equinus* complex; *SGSP Streptococcus gallolyticus* subspecies *pasteurianus*; *SGSG Streptococcus gallolyticus* subspecies *gallolyticus*; *SL Streptococcus lutetiensis*; *SISI Streptococcus infantarius* subspecies *infantarius*; *SE Streptococcus equinus*

*MALDI Biotyper was significantly more accurate in identifying the SBSEC isolates at the subspecies level compared with the VITEK2 (*P* = 0.010)

^†^There were no significant differences in the rates of correct identification of the SBSEC isolates at the species level between the MALDI Biotyper and the VITEK2 (*P* = 0.055)

^a^Identified with the highest score (score > 2.0)

^b^Identified with high probability (> 95%)

Details of “Low discrimination” are as follows

^c^SGSG (50%)/SGSP (50%) n = 1; SGSG (33%)/SGSP (33%)/SISI (33%) n = 1; SL (33%)/SGSG (33%)/SGSP (33%) n = 1; SL (51%)/SGSG (49%) n = 1; *Lactococcus raffinolactis* (34%)/SL (33%)/SGSG (33%) n = 1

^d^SL (51%)/SGSG (49%) n = 1; SL (50%)/SGSP (50%) n = 1

^e^SL (50%)/SISI (50%) n = 1

In contrast, the VITEK2 correctly identified 87% (34 of 39) of the SBSEC isolates at the species level and 67% (26 of 39) at subspecies level. The VITEK2 identified all the SGSGs at the subspecies level correctly. However, the VITEK2 identified 20 (69%) SGSPs with a high probability, five (17%) with low discrimination, four (14%) with no identification, and no isolate was misidentified. One SL isolate was misidentified as SGSP by the VITEK2.

The MALDI Biotyper was significantly more accurate in identifying SBSEC isolates at the subspecies level compared to the VITEK2 (92% vs. 67%, *P* = 0.010). In contrast, there were no significant differences in the rates of correct identification of SBSEC isolates at the species level between the MALDI Biotyper and the VITEK2 (100% vs. 87%, *P* = 0.055).

## Discussion

In the present study, SGSP (74%) was the dominant subspecies of SBSEC that caused bacteremia, followed by SGSG (13%), SL (10%), and SISI (3%). These results were consistent with those of previous studies [7, 19, 20]. Specific diseases have been found to be associated with specific SBSEC species or subspecies. For instance, SGSG bacteremia was found to be associated with infective endocarditis and colorectal cancer [4, 5]. In addition, SGSP and *S. infantarius* were found to be associated with biliary tract infections and biliary-pancreatic cancer [6–9]. In the present study, excluding primary bacteremia, infective endocarditis was the most common cause of bacteremia (26%), followed by biliary tract infections (23%). These results were also consistent with those of previous studies [8, 20]. However, there were no significant differences in the frequencies of the source of bacteremia among the SBSEC subspecies.

In the present study, there were no significant differences in the incidence of overall malignancies among each SBSEC subspecies. However, the incidence of overall colorectal cancer in patients with SGSG bacteremia was significantly higher than that in patients with bacteremia due to other SBSEC subspecies including SGSP. The strong correlation between colorectal cancer and SGSG has been well-established [5]. A systematic review and meta-analysis showed that the risk of acquiring colorectal cancer was lower in patients with SGSP than in those with SGSG at an odds ratio of 7.26, and that the incidence of colorectal cancer in patients with SGSP did not exceed the incidence in general asymptomatic individuals [5].

As many patients with SBSEC bacteremia have been identified as elderly with comorbidities [19, 20], it may have been difficult to perform colonoscopies in all the patients. In the present study, the primary bacterial sources in patients with colorectal cancer were infective endocarditis and primary bacteremia, and no patients had biliary tract infections. As the study used a retrospective design and no patients with biliary tract infection underwent colonoscopies, it is uncertain whether these patients had colorectal cancer. However, a previous large-cohort study found that colorectal cancer was less common in patients with biliary tract infections [6]. Therefore, in case of SBSEC bacteremia, patients with biliary tract infections may not require colonoscopies. Nevertheless, further studies are required to determine which patients with SBSEC bacteremia have a lower risk of colorectal cancer.

In previous studies, SGSP was associated with biliary pancreatic cancer [6–8]. In the present study, bile duct cancer was detected in three of 29 patients with SGSP bacteremia. Bile duct cancer was not found in patients with bacteremia caused by SBSEC, except in those caused by SGSP. Additionally, although 95% of the patients underwent CT scans, no patient was found to have pancreatic cancer. In each previous study, since the number of patients with biliary-pancreatic cancer and SGSP bacteremia was small [6–8], further investigation is required to clarify the association between SGSP bacteremia and biliary-pancreatic cancer.

In the present study, four patients with SL bacteremia were identified. The sources of SL bacteremia were infective endocarditis, biliary tract infections, and primary bacteremia. Since the incidence of SL bacteremia is less common than the incidences of SGSG and SGSP, only a few studies have investigated the clinical characteristics of SL bacteremia [19, 20]. Previous studies have reported infective endocarditis and biliary tract infections as sources of SL bacteremia [19, 20]. The results of the present study were consistent with those of previous studies, suggesting that the clinical presentation of patients with SL bacteremia is similar to that of patients with SGSG and SGSP.

In the present study, SGSP was identified in a pregnant woman with infective endocarditis. A previous study suggested that SGSP was an important pathogen of pregnancy-related infections leading to neonatal meningitis [21].

Further, the MALDI Biotyper was significantly more accurate in identifying SBSEC isolates at the subspecies level compared to the VITEK2. However, there were no significant difference in the rates of correct identification of SBSEC isolates at the species level between the MALDI Biotyper and the VITEK2. The MALDI Biotyper correctly identified all the SGSP isolates at the subspecies level. In 17 SGSP cases (59%), all the bacterial species identified using the MALDI Biotyper with a score > 2 were SGSP. Previous studies have also reported that the MALDI-TOF MS analyses using the MALDI Biotyper identified SGSP accurately [13, 22]. In contrast, the VITEK2 identified 69% of SGSP isolates at the subspecies level correctly. Eighty percent of the SGSG isolates were identified correctly at the subspecies level using the MALDI Biotyper. However, in all the SGSG cases, those with scores ≥ 2.0 obtained using the MALDI Biotyper included SGSP strains. In contrast, the VITEK2 correctly identified all the SGSG isolates with a high probability . The differences in the results of the MALDI Biotyper analysis may have been due to the number of reference strains of SGSP in the current database being greater than that of SGSG (7 strains vs. 4 strains). SL was more correctly identified at the subspecies level with the use of the MALDI Biotyper (75%) than with the VITEK2 (25%). SISI was misidentified with the use of the MALDI Biotyper as *S. equinus*. The same misidentification pattern was reported previously [13]. Moreover, the comparative evaluation of the two MALDI-TOF systems—MALDI Biotyper and VITEK MS—with the 16S rRNA and 16S–23S intergenic spacer region sequencing being used as the reference method, revealed several inaccuracies in both of the systems [13]. Additionally, the accurate identification with the MALDI-TOF MS was correlated significantly with the reference database for the target species [23]. Therefore, it is necessary to increase the number of reference spectra in the database for a more accurate identification of the SBSEC subspecies. The present study found that in terms of association between the subspecies and disease, colorectal cancer was associated significantly with SGSG bacteremia, while there were no significant differences in the frequencies of the source of bacteremia among the SBSEC isolates. The MALDI-TOF MS and phenotypic identification systems in the present and previous studies [13, 22] identified the SBSEC isolates at the species level accurately, but not at subspecies level. Additionally, it was difficult to identify SBSEC genetically using sequencing target genes such as *sodA* in clinical microbiological laboratories as a routine practice. Therefore, when SBSEC is detected in blood culture, it may be reasonable for clinicians to perform echocardiographies and colonoscopies to detect infective endocarditis and colorectal cancer in all patients with SBSEC bacteremia.

This study had some limitations. This was a retrospective study with a small sample size. The initial methods for identifying SBSEC differed between the three hospitals. There was an absence of a gold standard for the species identification of streptococci. Sequencing of *sodA* was performed only as an identification reference in the study. Additionally, in the MALDI-TOF MS analysis, we only analyzed the direct transfer method. Given the retrospective design of this study, 41% and 54% of the patients had undergone colonoscopy and transthoracic echocardiography, respectively, while only 8% underwent transesophageal echocardiography. Therefore, infective endocarditis and colorectal cancer may have been underestimated because not all patients underwent echocardiographies and colonoscopies. However, to the best of our knowledge, this was the largest study that investigated the incidence of SBSEC bacteremia in Japan. Further large-scale, prospective studies are required to clarify the clinical and microbiological characteristics of SBSEC bacteremia.

## Conclusions

We have reviewed the clinical characteristics of patients with SBSEC bacteremia retrospectively and evaluated the accuracy of identification of SBSEC isolates using the MALDI-TOF MS and phenotypic identification systems, with *sodA* gene sequencing as a reference. While the source of bacteremia was similar between each SBSEC subspecies, colorectal cancer was associated significantly with SGSG bacteremia. The MALDI-TOF MS was significantly more accurate in identifying SBSEC isolates at the subspecies level compared with the phenotypic identification systems. The accurate distinction between the SBSEC subspecies using the MALDI-TOF MS and phenotypic identification systems remains difficult. However, the MALDI-TOF MS and phenotypic identification systems can accurately identify SBSEC at the species levels. Therefore, when SBSEC is detected in a blood culture, it may be reasonable for clinicians to perform echocardiography and colonoscopy to investigate infective endocarditis and colorectal cancer.

## Data Availability

The dataset analyzed in this study is available from the corresponding author upon reasonable request.

## Abbreviations

SBSEC: *Streptococcus bovis*/*Streptococcus equinus* Complex
SGSG: *Streptococcus gallolyticus* subspecies *gallolyticus*
SGSP: *Streptococcus gallolyticus* subspecies *pasteurianus*
MALDI-TOF MS: Matrix-assisted laser desorption/ionization time-of-flight mass spectrometry
SL: *Streptococcus lutetiensis*
SISI: *Streptococcus infantarius* subspecies *infantarius*
